# Ask the experts: Community Engagement studios to inform research on cannabis use in cancer symptom management

**DOI:** 10.1017/cts.2025.10

**Published:** 2025-01-30

**Authors:** Brittney Greene, Grace Mckenzie, Keenan Gibbons, Salimah H. Meghani, Brooke Worster, Rebecca L. Ashare

**Affiliations:** 1 Department of Psychology at the State University of New York, University at Buffalo, Buffalo, NY, USA; 2 Clinical and Translational Science Institute, Jacob’s School of Medicine, State University of New York, University at Buffalo, Buffalo, NY, USA; 3 School of Public Health and Health Professions, University at Buffalo, Buffalo, NY, USA; 4 University of Pennsylvania School of Nursing, Philadelphia, PA, USA; 5 Sidney Kimmel Cancer Center, Thomas Jefferson University, Philadelphia, PA, USA

**Keywords:** Community engagement, cannabis, health disparities, cancer, community experts

## Abstract

**Aim:**

Despite one-third of patients with cancer using cannabis for symptom management, little is known about their access to and usage of cannabis. Community Engagement (CE) studios involving community experts with chronic health conditions were used to inform a qualitative study on access to and use of cannabis products among patients with cancer.

**Method:**

We conducted two 2-hour CE studios with residents from Western NY (WNY) (*N* = 18). Our sample primarily included White and Black residents (56% vs. 39%). After a researcher-led 10-minute presentation, a community facilitator led the discussion, which focused on questions about challenges to cannabis use, recommendations for providers when discussing cannabis with patients, and community factors influencing use.

**Results:**

Community experts reported that state legalization of cannabis made it easier to access cannabis, but the costs of cannabis and distance to dispensaries hindered their ability to obtain it. Discrimination was also a key barrier to medical cannabis receipt. There were differences in the perceived safety of where to obtain cannabis (dispensaries vs. friends). Community experts wanted providers to be more informed and less biased about recommending cannabis. Community experts recommended conducting focus groups for the subsequent study to ask questions about cannabis use.

**Conclusion:**

The CE studios encouraged us to switch formats from qualitative interviews to focus groups and provided guidance on question topics for the subsequent study. Incorporating community expert’s feedback through CE studios is an effective strategy to design more impactful studies.

## Introduction

With the growing legalization of cannabis in the USA and its purported medicinal effects to alleviate cancer-related symptoms, it is imperative to contextualize factors regarding its use among patients with cancer [1]. Approximately one-third of patients with cancer use cannabis to treat cancer-related symptoms such as pain, mood, sleep, and appetite [2–5]. However, there is limited data regarding the pattern of cannabis use, methods of use, and perceived risks and benefits of its use. Furthermore, discussions surrounding cannabis use may be controversial as cannabis is still classified as a Schedule I drug by the US Food and Drug Administration, and the legality (and therefore accessibility) of cannabis use varies by state [6].

This paper aimed to receive community input on how to facilitate conversations in a subsequent research study among patients with cancer who primarily reside on the East Coast. Our goal was to better understand the barriers that may emerge for this population when talking with researchers about cannabis use and identify ways to attenuate these barriers in a future study in which we planned to conduct qualitative interviews among patients with cancer regarding their cannabis use. The main objective of this paper was to explore factors and barriers linked to cannabis use among residents with similar characteristics as our future sample (e.g., chronic condition, cannabis use). The Western NY (WNY) region reflects many of the structural and systematic barriers to cannabis use for several reasons. First, adult recreational and medicinal cannabis use was legalized in 2021 in the state of New York, which makes it easier to identify barriers (e.g., lack of access to dispensaries and stigma) to cannabis use other than regulatory barriers. Second, WNY is an economically and racially diverse area, which are both important factors in cannabis access.

Several studies indicate that low-income, marginalized, and rural groups encounter numerous structural barriers that hinder their access to equitable treatment options [7–9]. Although there may be some commonalities, such as lack of health insurance coverage, discrimination, and limited health literacy among these groups who typically reside in deprived neighborhoods, it is crucial to recognize that the specific barriers contributing to these disadvantages may vary [10]. For example, medical cannabis services may be more commonly found in rural and urban areas, but the distance to these services may differ based on access to transportation within these areas [11]. Hence, it is essential to understand the diverse challenges faced by these groups in accessing cannabis. This information will be used to shape the questions about cannabis use for our subsequent research study. Additionally, we aimed to gather input from the community on ways to enhance the design for the future study. Due to few studies examining cannabis use among patients with cancer with a qualitative component, we adopted an approach to elicit feedback from the community on the questions to ask in our upcoming study and best practices when asking patients with cancer about their cannabis use.

Community Engagement (CE) studios are a national consultative approach where stakeholders (community experts; typically having similar characteristics as the study population) provide feedback to researchers about their study design, measures, and recruitment [12]. CE studios include a brief 10-minute presentation conducted by researchers to summarize the research questions, followed by a conversation with community experts guided by a trained facilitator (the researchers are present but are not active during the discussion). The community experts share their experiences and opinions in response to the questions to be addressed [12]. In their study of 28 post-CE studio evaluations, Joosten et al. (2015) found that CE studios had several benefits, including increasing the researcher’s understanding of and sensitivity to the study populations, improving the feasibility of the projects, and informing plans on dissemination. By involving the community, CE studios can increase the quality and relevance of research [13].

The use of CE studios is emerging in several areas of research and populations, including diverse and rural populations [14–19]. CE studios have assisted researchers in adapting their studies by modifying and creating educational content, increasing the availability of days and times for intervention sessions, and covering transportation costs for future participants [14,17]. CE studios have also built trust and respect between researchers and community experts from historically excluded populations [20].

Several studies among patients with cancer have demonstrated that community engagement strategies can promote positive effects on research participation such as reducing attrition and implementing collaborative approaches between the study participants and the researchers [21–25]. However, only one study that we know of utilized a CE studio to inform research on patients with cancer. Skiba et al. (2024) used a CE studio to inform an exercise program among Hispanic men with prostate cancer. They modified the exercise intervention (Exercising Together) to be more culturally responsive (e.g., available in English and Spanish) and less burdensome for the participants. To our knowledge, no study – in any population – has used CE studios to inform research surrounding cannabis.

Increasing community involvement through CE studios could help researchers conduct participant-centered research while being mindful of participants” challenges and appreciating their willingness to participate in research studies on sensitive topics.

## Methods

### Studio design

**Figure 1.**
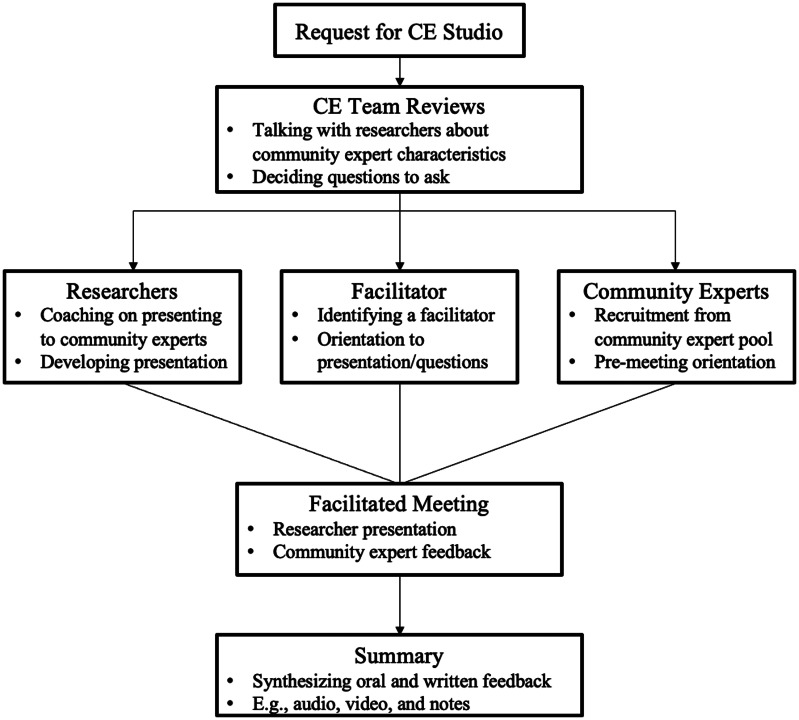
Community Engagement studio procedure. *Note*. The figure models the community engagement studio process and is an adaptation from Stock et al. (2022).

Two 2-hour CE studios were conducted, one in-person and the other delivered via Zoom. Local community experts preferred an in-person format. However, providing a virtual format increased accessibility to residents in rural WNY. Both sessions were audio-recorded. CE studios followed the format described by the Meharry-Vanderbilt Community-Engaged Research Core [12]. CE studios are not considered research. Rather, a CE studio is a method of communication with a desired community for important feedback to design a study, project, or other research. The CE studio process is described in Figure 1. In both studios, researcher BG, who is a graduate student in psychology, conducted a 10-minute presentation to community experts about the research study, including background information on cancer and its treatment (e.g., prevalence, opioids, and racial disparities), the growing legalization of cannabis, and the methods of the upcoming study. The presentation was developed by the research team (RA, who is the primary investigator of the study and BG) and community engagement specialists (GM and KG, who have Masters in Education or Public Health and coordinated nine CE studios). Each studio had different trained facilitators who had similar characteristics as the community experts and over a decade of experience involving community engagement. Facilitators guided the conversation around three key questions: 1) What are the primary challenges in obtaining cannabis? 2) What should providers consider/avoid when asking patients about cannabis use? 3) What community factors influence decisions to use cannabis? Several additional questions were asked to inform and/or modify the future study design to increase participant engagement. Researchers asked questions sparingly as recommended by the Meharry-Vanderbilt Community Engaged Research Core, which emphasizes the role of researchers as the audience learning from the community experts [27]. A full description of the questions is listed in Table 1. Facilitators were compensated $250, and community experts were compensated $50 (provided after the CE studio for in-person and mailed out to those participating in a virtual CE studio). Community experts participating in an in-person CE studio were also provided a meal. CE studios are exempt from human subjects research due to the consultative study design and thus do not require Institutional Review Board approval [27].

**Table 1.** Community Engagement (CE) studio discussion questions

1. What are you hearing about cannabis in your community?
2. What are the primary challenges you have in obtaining cannabis?
3. How comfortable do you feel about talking about cannabis with different people (e.g., family, friends, doctors)?
4. Are there any community factors that could influence your decision to use cannabis?
5. What are some benefits and risks of cannabis use?
6. What types of questions should we ask in this study?
7. How should we engage patients in this type of study?
8. Do you think people with cancer would take part in this study? Why?
9. Do you think that patients would prefer to meet in a group like this one or one-on-one with a researcher?

### Recruitment

All community experts were recruited through the Buffalo Research Registry (BRR) and the Community Engagement Core listserv. The BRR is a database accessible to people living in WNY interested in participating in research at the University at Buffalo (UB). Flyers created for the study’s CE studios were sent to BRR and distributed to potential participants. The Community Engagement Core listserv includes several community partners, organizations, and agencies that are members of the Clinical and Translational Science Institute Community Core at UB. The study flyer was sent to these members and disseminated to their staff and clients. Efforts were made to recruit community experts from a wide geographical area in WNY, including urban, suburban, and rural areas. The Community Engagement Core listserv aided in recruiting several community experts, particularly from suburban and rural communities. Eligibility criteria included being 18 years or older, using cannabis or having an interest in using cannabis, and having a chronic condition or extensive knowledge of cancer (e.g., as a caregiver or provider).

### Procedure for identifying topics

Audio recordings and extensive notes taken during the studios were consolidated, and a synopsis was developed for each studio by (KG and GM). Members of the CE studio team (KG, GM) and research team (BG) reviewed the notes and audio recordings taken during the studios, checked the transcripts, and compared the information for accuracy. The data were coded based on CE studio questions and responses from community experts. Data were analyzed in Excel and stored within a box drive under the University at Buffalo, which was only accessible to the study team. Data were evaluated using COREQ guidelines for comprehensive reporting [28].

## Results

### Community expert characteristics

A total of 18 community experts were included. Community experts included residents from mid-sized urban metro areas (*N* = 14), suburban areas (*N* = 3), and rural areas (*N* = 1) in WNY. These classifications were provided based on the Urbanization Perceptions Small Area Index [29]. The in-person CE studio primarily consisted of residents from urban and suburban areas, whereas the virtual studio consisted of residents from suburban and rural areas. All community experts had chronic conditions (e.g., diabetes, lupus, HIV, chronic pain, and cancer). Approximately 72% of community experts were currently using or have used cannabis, and 28% were interested in using cannabis. The sample was predominately White or Black (56% and 39%, respectively), which is comparable to percentages in WNY cities like Buffalo (47% and 33%, respectively) [30], and female (72%). The average age was 49 years. Demographic characteristics are further described in Table 2.

**Table 2.** Demographic characteristics among community experts

Characteristics	Community experts (n = 18)
Age mean (range 22–73)	49.1
Sex n (%)	
Male	5 (27.8%)
Female	13 (72.2%)
Race n (%)	
White	10 (55.6%)
African American/Black	7 (38.9%)
More than one race	1 (5.6%)
Cannabis use n (%)	
Using/past use	13 (72.2%)
Interested in using	5 (27.8%)
Chronic condition n (%)	
Cancer	2 (11.1%)
Autoimmune disease	4 (22.2%)
Chronic pain	2 (11.1%)
Diabetes (unspecified)	2 (11.1%)
HIV	1 (5.6%)
Mood disorders	4 (22.2%)
Other	3 (16.7%)

*Note*. Chronic condition frequency counts include community experts with multiple conditions.

### Overview of the findings

Responses from community experts revealed several sub-topics related to our study questions and primarily focused on barriers to cannabis use. These correspond to 1) Patient-Level Factors (Stigma & Fear of Opioids, Discrimination, Side Effects and Benefits, and Lack of Resources & Knowledge), 2) System-Level Factors (Cost of Cannabis & Location, Safety/Regulation of Products, and Provider Considerations), and 3) Legal Factors (Legalization of Cannabis, Differences between Federal & State Laws). Key quotes are reflected throughout the findings as shown in Table 3.

**Table 3.** Additional community expert quotes

	Topics	Community expert quotes
Patient-level factors		
	Stigma & fear of opioids	
		“I don’t want [opioids], they make me scared… you hear so many things about them.” (Community Expert – Urban WNY, Female, 36)
	Discrimination	
		“It is a disproportionate [fewer] number of minorities in dispensaries [pertaining to having a medical cannabis card].” (Community Expert – Urban WNY, Female, 57)
System-level factors		
	Safety/regulation of products	
		“Who knows what you’re getting now [in response to street cannabis]?” (Community Expert – Urban WNY, Female, 59)
	Provider considerations	
		“I never hear my doctor ask me once about what I think.” (Community Expert – Urban WNY, Female, 57)
		“Tell me about the medical facts, your knowledge and expertise, NOT your opinion.” (Community Expert – Urban WNY, Female, 53)
		“We’re not cattle, we need to be taken care of” (Community Expert – Urban WNY, Female, 57)
Legal factors		
	Legalization of cannabis	
		“It is something I would have never thought to use… now that I cannot be drug tested, absolutely.” (Community Expert – Rural WNY, Female, 53)
	Differences between federal & state laws	
		“It’s still illegal…people who receive federal welfare programs and housing cannot safely use even medical cannabis inside their home. (Community Expert – Rural WNY, Male, 29)

### Patient-level factors

Community experts identified several patient-level factors that influence their decision or interest in using cannabis. These factors include perspectives on cannabis and opioids, the potential impact of discrimination on access to cannabis, and the consideration of benefits and risks associated with cannabis use. Furthermore, many community experts expressed a need for more evidence-based information to guide their decisions regarding cannabis use.

### Stigma & fear of opioids

Community experts had supportive discussions with family and friends about cannabis use. They felt that their loved ones’ perspectives did not heavily influence their decisions to use. However, two community experts reported that they did not discuss the details of their cannabis use with their loved ones due to perceived judgment. Another community expert mentioned that they only shared their use of CBD products and not THC-infused products with their partner because of perceived non-acceptance. Several community experts indicated that their decision to use cannabis was partly driven by their perception that cannabis was more socially acceptable than opioids. There was also consensus regarding negative perceptions of opioids, with quotes such as “Opioids, I think, are much less approved by the populace” (Female, White, 70). Community experts also noted that the language used to describe cannabis has evolved (e.g., marijuana/pot to cannabis), making it more widely acceptable to discuss in medical contexts. One community expert considered how stigma may impact patients with cancer, suggesting that patients with cancer may not want to use cannabis because they are already stigmatized for their condition, potentially facing double stigma if they choose to use cannabis.

### Discrimination

Some community experts residing in urban and suburban WNY reported discrimination being a challenge to accessing cannabis. Several experts of color indicated that belonging to a marginalized group may make it harder to access medical cannabis. One expert discussed medical cannabis cards, which are state-issued cards that identify qualified patients who can legally use cannabis for medical purposes. They reported that Black patients with chronic conditions appear less likely to receive medical cannabis cards and thus receive their cannabis from dispensaries at disproportionately lower rates than their White counterparts.

### Side effects & benefits

Community experts reported several benefits and risks associated with using cannabis. Benefits included pain relief, relaxation, improved appetite and mood, and decreased anxiety and nausea. One community expert said, “It helps me get through the day. Takes the edge off and makes me feel wonderful” (Male, White, 73). Two community experts reported using cannabis as an alternative to opioids and anti-inflammatory medication. Some community experts reported experiencing side effects of cannabis including having a “bad trip,” increased heart rate, fatigue, and heightened anxiety and pain. Two community experts expressed concerns about potential lung issues from smoking cannabis. Community experts also indicated that cannabis may have increased side effects when used with prescription drugs. Given these risks, some community experts modified their use by choosing strains to maximize benefits and minimize harms and opting for edibles over inhaled forms.

### Lack of resources & knowledge

Community experts reported that they learned about cannabis from their parents, relatives, and adolescent peers. They also mentioned that they continue to learn new information about cannabis from the younger generation. For example, a young community expert mentioned educating their mother about the perceived benefits of cannabis and encouraging her to try it. Another community expert stated, “My adult children are teaching me now… we need to keep up with the younger generation” (Female, Black, 53). Additionally, community experts mentioned that they relied on internet searches for information about cannabis. However, they found that the information available online was conflicting, making it confusing to understand the different ways to use cannabis. Many experts, particularly those living in suburban and rural WNY, expressed that they were unaware of all the available options for obtaining cannabis in their area.

### System-level factors

Community experts pointed out several systematic barriers that affect their decision to use cannabis and the methods of using it. These barriers include the increasing cost of cannabis, the distance from dispensaries and Native American reservations, as well as concerns about the safety and regulation of cannabis. They also mentioned that communication with healthcare providers is a major obstacle to accessing cannabis and provided recommendations for providers to facilitate trust with patients.

### Cost of cannabis & location

Community experts highlighted that the cost of cannabis, in combination with location, is a major deterrent to its use. For example, some experts in urban WNY noted how the costs of cannabis have risen substantially, particularly within dispensaries. One expert in urban WNY stated, “Dispensaries are usually located in the suburbs… so not only is it a monetary factor to purchase [cannabis] when you get there, you have to get there” (Female, Black, 57). Similar sentiments were expressed among community experts in suburban and rural WNY, with some individuals purchasing cannabis at Native American reservations due to the lower costs compared to dispensaries. One community expert expressed, “If you are in a financial situation, you are going to the reservation 100% of the time. The cost of products at legitimate dispensaries in NYS is prohibitively expensive to your average medical and recreational user” (Male, White, 29).

Many community experts in urban WNY reported difficulties in obtaining medical and recreational cannabis from dispensaries primarily due to distance. They said that dispensaries are mainly located in the suburbs, which are not easily reachable by public transportation. Only two experts in this area reported being able to get medical cannabis regularly from dispensaries, while community experts in suburban and rural WNY stated that dispensaries were too far away to access frequently. As a result, community experts largely relied on local sources such as getting cannabis from a dealer, a friend/relative, or visiting a Native American reservation. However, one community expert in urban WNY reported that reservations were also inaccessible, whereas the majority of community experts residing in suburban, rural, and some areas in urban WNY obtain their cannabis from reservations.

### Safety/regulation of products

Community experts discussed the potential hazards of obtaining cannabis in their community. Community experts in urban WNY expressed concerns about buying cannabis on the street due to the possibility of it being mixed with dangerous substances like fentanyl. One community expert stated, “It’s better now to get it from a dispensary. They put so much stuff in it when you get it off the streets” (Female, Black, 57). Some community experts had concerns about the safety of obtaining cannabis from Native American reservations. Two community experts reported experiencing adverse side effects due to incorrect dosage instructions from an employee at a cannabis outlet on a reservation, “Cannabis is really not regulated. We were told to take at least three squares of [edible]. I wound up having to call poison control” (Female, White, 70). With the discussion of safety, trust emerged as a prominent factor. Several community experts in urban WNY expressed buying from a trusted dealer, relative, or friend, whereas others residing in different areas of WNY reported refusing to buy from a dealer because of safety concerns.

### Provider considerations

Community experts in urban WNY communities discussed potential racial bias among medical providers. Some reported that providers assume marginalized and low-income groups cannot afford medical cannabis and thus do not offer it as a treatment option. They also reported that providers perceive the pain of Black patients with chronic conditions as less severe compared to their White counterparts: “Black people experience pain just like white people do” (Female, Black, 57) and “There are some things we can use, but the doctors seem to think only about one group” (Female, White, 36). Additionally, many community experts felt that providers are primarily concerned about profit and prestige, which leads them to overprescribe opioids and not offer alternatives such as medical cannabis. One community expert stated, “They are doing it for their own name, status, or money” (Female, White, 54). Considering these negative experiences, community experts suggested that healthcare providers should build trust with patients by creating a safe environment for patients, minimizing their implicit biases, and supporting their patients” decisions. Additionally, they suggested that providers learn more about cannabis and initiate conversations about cannabis use.

### Legal factors

Community experts expressed mixed opinions about the legalization of cannabis and its implementation in NY state.

### Legalization of cannabis

Although the majority of community experts stated that legalization did not change their perspective on cannabis, they noted that legalization has made cannabis more accessible. One community expert stated that legalizing cannabis for both medicinal and adult use has significantly reduced barriers for them. Another community expert reported using cannabis only after it became legal to comply with the laws and regulations. Additionally, some community experts expressed interest in cannabis after it became legal due to concerns about workplace drug testing.

### Differences between federal & state laws

Even though cannabis is legal for both medicinal and adult use in New York, it is still illegal at the federal level and community experts noted that there is still uncertainty about whether cannabis possession and use can lead to institutional and/or legal consequences. One community expert expressed concerns about using cannabis on their college campus, as it may violate campus policies. Another community expert pointed out that conflicts between federal and state policies regarding cannabis use can have serious consequences. Specifically, they highlighted that due to its Schedule I status federally, individuals living in federally subsidized housing could face eviction if caught using or in possession of cannabis.

### Feasibility & recommendations

All community experts indicated that patients with cancer would be interested in participating in a study about their experiences with cannabis use including pain management, bodily autonomy, and healthcare. They also mentioned that patients with cancer who vary in cannabis frequency, methods, and receipt (e.g., dealer, dispensary, reservation) should be included. The experts suggested having multiple formats, including virtual sessions, to increase access for patients. They also suggested that a group format (vs. one-on-one interviews) would allow for more open discussion, shared learning, and increased comfort. Specifically, many community experts openly shared their experiences with cannabis use to manage symptoms, which likely stemmed from knowing the group shared these similarities. Furthermore, the group setting allowed community experts to hear new and different perspectives which enabled them to think further about their own experiences. For instance, one community expert reported interest in cannabis use, but not wanting to smoke it. In response, other community experts discussed alternative methods (e.g., edibles). Community experts were open to discussing topics related to access to cannabis use, stigma, and provider considerations, suggesting these topics should be explored in our study. The community experts also indicated that providing resources such as a “How-to” sheet for getting a medical cannabis card, educational and support group resources about cannabis use, and having a provider or researcher present to answer questions about cannabis use would be helpful. For recruitment and engagement, community experts recommended contacting providers and partnering with cancer centers in outreach events.

## Discussion

Cannabis is emerging as a potential treatment option to manage cancer and cancer treatment-related symptoms. The broader goal of this research is to better understand how patients make decisions about accessing cannabis, including where they obtain it, which products they use, and how this may relate to symptom management. Given the limited research on factors associated with cannabis access and how accessibility may impact patient-reported outcomes, we utilized CE studios to inform the study design. Community experts provided important insight regarding which questions to ask patients with cancer about their cannabis use and best practices when asking these questions and guided our decision to modify the study design.

A key outcome of these CE studios is the preference for focus groups versus qualitative interviews. We initially planned to conduct qualitative interviews to gather comprehensive information while maintaining a greater sense of privacy and comfort in sharing their experiences. However, based on feedback from community experts, we are now considering implementing focus groups rather than qualitative interviews. Focus groups are a systematic research approach for collecting data from multiple participants [31]. Community experts believe that focus groups may be a better method for engaging patients compared to qualitative interviews due to several advantages. First, focus groups can promote a sense of camaraderie among participants and enhance their willingness to share their experiences [32]. Due to the mistrust between researchers and patients, patients may not have felt as comfortable discussing their cannabis use one-on-one, potentially limiting the information we could gather, making focus groups a possible effective option. Indeed, community experts emphasized the significance of shared learning. The group format provided the opportunity for community experts to discuss their experiences about cannabis with less consensus and increased comfort discussing this topic since all members were past or current cannabis users or interested in cannabis use, which may reduce stigma. Second, focus groups can reduce the burden on researchers by collecting data from multiple participants at once, lessening the pressure on any single participant [32]. Indeed, new topics and themes may emerge, which was observed by the natural conversations and varying perspectives that emerged during the CE studios, which is a strong advantage of group discussion.

Our CE studios involved residents from various parts of WNY, which allowed for data about cannabis use sources and racially and economically diverse experiences regarding obtaining cannabis, making it a key region to explore cannabis access. For instance, some community experts obtained cannabis from Native American reservations, which provides another option for obtaining cannabis, particularly for those residing in rural regions. Additionally, Native American reservations have differing state regulations than dispensaries, which may capture variability in access (e.g., density of Native American reservations vs. dispensaries, availability of cannabis products) and potential benefits and risks of using cannabis products from Native American reservations compared to dispensaries. Furthermore, having a diverse group uncovered several systematic and structural barriers to obtaining cannabis despite the recent growth of cannabis sources such as dispensaries. CE studios were conducted in both in-person and virtual formats. This approach increased the participation of community experts and allowed us to gather diverse perspectives based on different locations. As a result, our focus groups will offer both in-person and virtual options

The community experts shared their experiences regarding barriers to cannabis use, factors affecting their use, and their opinions on discussing cannabis use with medical providers. We found common topics across CE studios, such as challenges in accessing dispensaries, improved access following the legalization of cannabis in New York, and the desire for medical providers to be more knowledgeable about cannabis use. We also found differences across community experts in where they obtained cannabis and their perceptions of discrimination as a barrier to cannabis use. Recognizing challenges to cannabis use, including discrimination, will be crucial to address in our larger project, especially considering our recent finding that Black patients with cancer may be less likely to have medical certification for cannabis [33], as well as findings from other studies revealing that Black patients are less likely to receive treatment options such as opioid prescriptions as a result of cancer and its treatment when compared to their White counterparts [34]. There is limited data on how experiences of discrimination among patients with cancer may hinder efforts to obtain cannabis, making it an essential question to explore in the upcoming study. Building on these insightful discussions, our questions to patients will focus on these topics, including the topic of discrimination.

### Limitations

We need to consider the study’s limitations. The upcoming study for which these CE Studios were conducted will include Black and White patients with cancer from the East Coast primarily residing in New York or Pennsylvania. Our CE studios only included community experts from WNY. Pennsylvania’s laws regarding cannabis only include medical cannabis, not adult use like New York. It is possible that patients in Pennsylvania may have different perspectives than those in New York. Furthermore, findings from this study may not be generalizable to other countries where cannabis regulations differ or who have more homogeneous populations in which some of these barriers may not be as prevalent. Additionally, our sample size of community experts was similar to previous CE studios [18,35], but given the larger implications for the future study, it would have been helpful to hear more perspectives on cannabis use from residents of suburban and rural WNY.

## Conclusion

Incorporating community expert feedback through CE studios is an effective strategy to inform study design and promote participant engagement. Our CE studios revealed that using a group format to ask questions about cannabis use may increase participant comfort and allow for a more efficient means of collecting qualitative data. Community experts suggested having both in-person and virtual formats and providing resources to address participant concerns. Importantly, community experts felt comfortable discussing issues related to cannabis access and were candid when describing decisions around cannabis use. These CE Studios will enhance our research by ensuring that community voices are reflected in the format and content of the questions we ask.
